# Supplementary figures and images for: Meta-analysis of outcomes from drug-eluting stent implantation in femoropopliteal arteries
Source: PLoS One. 2023 Sep 21;18(9):e0291466. doi: 10.1371/journal.pone.0291466 (PMC10513203; doi:10.1371/journal.pone.0291466)

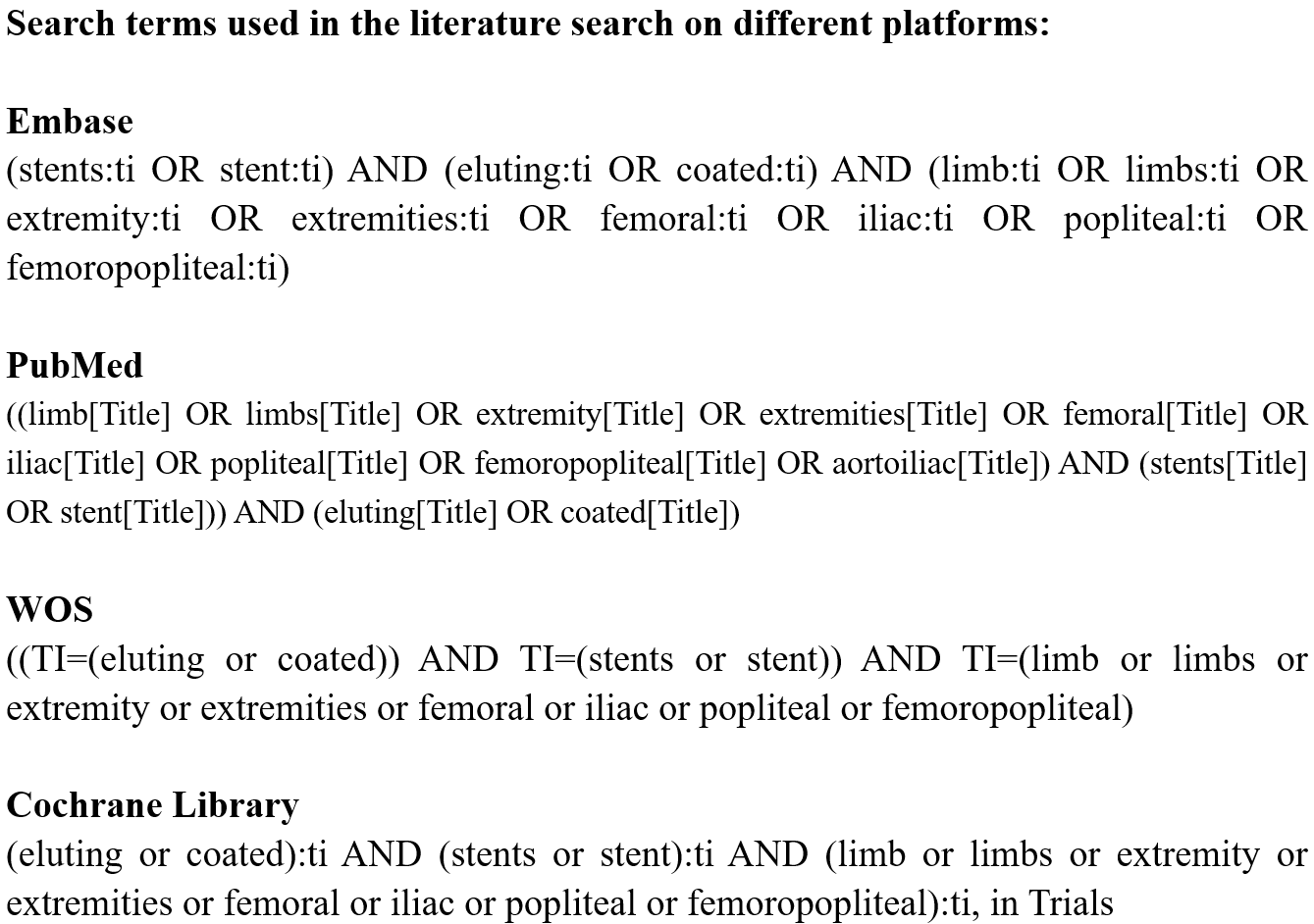

Supplement: S1 Fig — (TIF) [file pone.0291466.s002.tif]

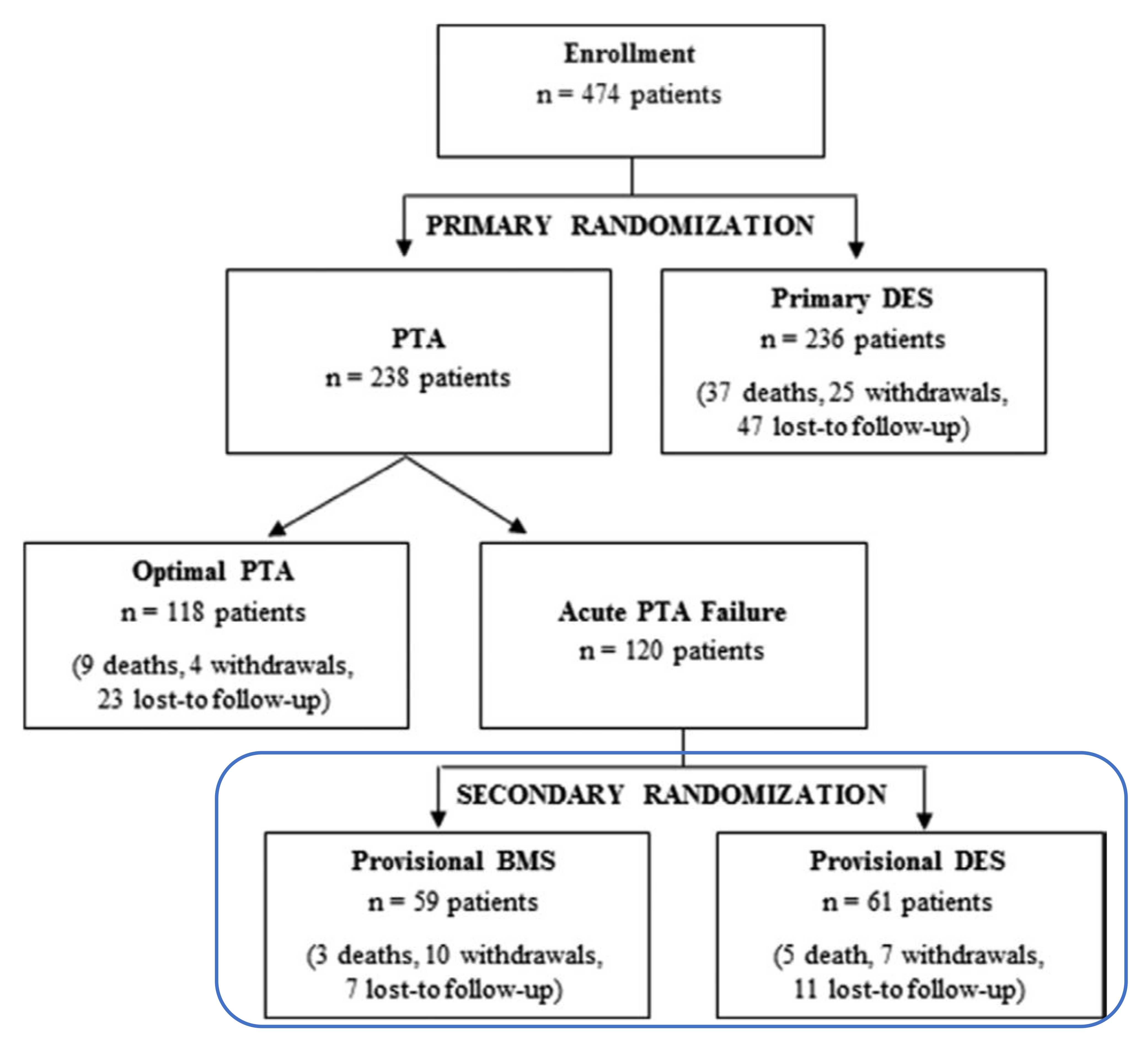

Supplement: S2 Fig — (TIF) [file pone.0291466.s003.tif]
